# Public health round-up

**DOI:** 10.2471/BLT.15.010615

**Published:** 2015-06-01

**The end of Ebola in Liberia**

Liberians celebrate the end of the Ebola virus disease outbreak in the west African country on 9 May. The news came as an independent panel released a report with its assessment of WHO’s response to the Ebola outbreak. The report was due to be discussed at the World Health Assembly from 18 to 26 May. http://www.who.int/csr/resources/publications/ebola/ebola-interim-assessment/en/

**Figure:**
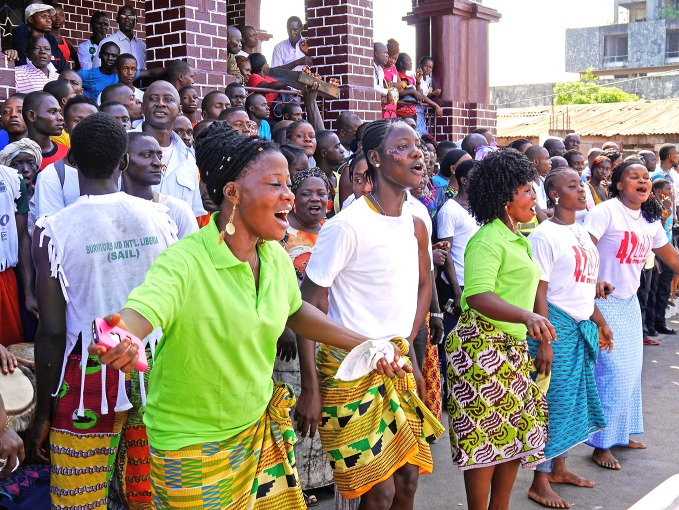
WHO/M. Winkler

## Nepal health emergency

WHO is coordinating the response to the health emergency with the Nepal government following the devastating 7.8 magnitude earthquake that struck the Himalayan kingdom on 25 April, killing more than 8000 people and injuring more than 18 000 (as of 12 May).

Repeated aftershocks, including another major earthquake registering 7.3 on the Richter scale on 12 May, have also been recorded, putting the lives and health of millions of people at risk in many of the country's more than 70 districts.

Twenty-six hospitals and some 900 health facilities – mainly small health posts in remote, mountainous communities – were damaged or destroyed by the quake.

The five major hospitals in the capital Kathmandu continued to function thanks to retrofitting and other risk-reduction measures as part of a national preparedness campaign for a major earthquake.

More than 50 000 patients had been treated in hospitals in the 14 districts that were most affected by the earthquake as of 5 May, but gaps remained across many parts of the country in both emergency and routine health-care provision.

The Nepalese government and WHO have called on governments and other donors to provide health-care workers through two systems: the United Nations Health Cluster that provides humanitarian care workers and WHO’s foreign medical team initiative.

“Nepal’s earthquake increases risks for patients suffering from a range of diseases. The disruption of medication, especially for major diseases such as diabetes and hypertension, can be very dangerous and even life-threatening,” said WHO medical officer Dr Frank Paulin, one of 20 emergency response staff sent to Nepal within days of the disaster.

WHO has contributed more than US$ 1.1 million to the emergency response operations in Nepal and sent medicines, emergency medical kits and other health supplies to treat tens of thousands of people.

 “Major challenges remain, including securing enough funding for the country’s health sector given the risk of more aftershocks and the rainy season, which starts soon, as that increases the risk of outbreaks of diarrhoeal diseases, acute respiratory infections and other diseases,” said Dr Roderico Ofrin, WHO regional coordinator for health security and emergency response.

http://www.who.int/emergencies/nepal/en/

## Antimicrobial resistance

Only 34 of 133 countries that responded to a WHO survey in 2013–2014 said they had comprehensive national plans to fight resistance to antibiotics and other antimicrobial medicines and even fewer already have systems in place to combat the problem.

The survey findings are presented in a new report entitled *Worldwide country situation analysis: response to antimicrobial resistance* that was released on 29 April.

It is the first WHO survey of governments’ own assessments of their response to resistance to antimicrobial medicines that are used to treat conditions such as bloodstream infections, pneumonia, tuberculosis, malaria and HIV infection.

Poor laboratory capacity as well as weak infrastructure and data management are barriers to effective surveillance systems capable of detecting antimicrobial resistance, identifying trends and monitoring for outbreaks, the report found.

Antibiotics and other antimicrobial medicines are sold without prescription in many countries. Many countries also lack standard treatment guidelines, increasing the potential for overuse of antimicrobial medicines by the public and medical professionals, it said.

The report found that public awareness of the issue was low in all six WHO regions and that many people still believe antibiotics can cure viral infections, such as the common cold, despite public information campaigns.

Another major problem is the lack of programmes to prevent and control hospital-acquired infections.

The report reiterated the need for all countries to have in place a fully funded, comprehensive national plan to address antimicrobial resistance across all sectors. It stressed the need, in particular, for more education and collaborative awareness-raising campaigns, appropriate regulations and standards, and capacity building in the area of monitoring and surveillance.

The report comes a year after WHO’s first report on the extent of antimicrobial resistance, which warned of a post-antibiotic era in which much of modern medicine could become impossible.

“Much more work needs to be done to combat one of the most serious global health threats of our time,” said Dr Keiji Fukuda, WHO Assistant Director-General for Security.

http://www.who.int/drugresistance/documents/situationanalysis/en/

**Cover photo**

Thousands of people were killed or injured in the earthquake that hit Nepal on 25 April. This child was rescued with her mother from the ruins of their home in Gorkha district and taken to the Nepali Army Base Camp in Kathmandu to receive medical care.

**Figure:**
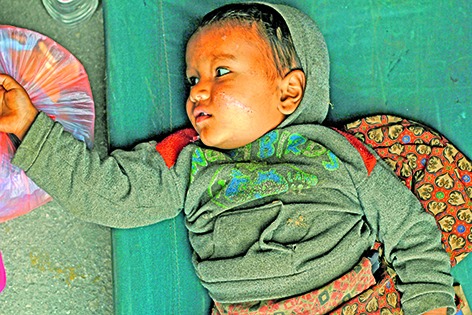
WHO/Ahmer Khan

## Goodbye MDGs

Improvements in human health have been mixed during the 25 years since 1990, the baseline for measuring the United Nations Millennium Development Goals (MDGs), according to an analysis in *World health statistics* 2015 released last month.

In September, countries are due to gather at the United Nations General Assembly in New York to decide on new global development goals that will replace the MDGs, which end this year.

“The MDGs have been good for public health. They have focused political attention and generated badly needed funds for many important public health challenges,” said Dr Margaret Chan, Director-General of WHO.

“While progress has been very encouraging, there are still wide gaps between and within countries,” she said.

Progress has been made in making water safer to drink, the analysis shows. The global target to halve the proportion of people without access to improved drinking-water sources in 2010 has been met, but countries in the WHO African and Eastern Mediterranean Regions are lagging behind.

The analysis also noted progress in improving child nutrition and reducing child mortality as well as reducing the incidence of malaria, tuberculosis and HIV infection.

But the world is unlikely to meet the target on “access to improved sanitation” at the end of this year. Around 1 billion people lack access to toilets and must defecate outdoors.

Substantial progress was made during the 25 years in reducing maternal mortality. The proportion of women who die from the complications of childbirth was reduced by 45% – nearly half – globally, although this fell far short of the ambitious target of 75%.

The proportion of deliveries by skilled birth attendants, including midwives or doctors, increased to 74%, short of the 90% target, the analysis showed.

The *World health statistics* is an annual compilation of key health statistics from WHO’s 194 Member States.

http://www.who.int/gho/publications/world_health_statistics/2015/en/

## Naming diseases 

WHO has issued guidance on the naming of new infectious diseases to minimize possible negative impacts on trade, travel, tourism or animal welfare and avoid causing offence to any cultural, social, national, regional, professional or ethnic groups.

The names of diseases are formally determined by the International Classification of Diseases (ICD) and there is a well-established taxonomy and nomenclature of pathogens. The new guidance does not propose any change to these, but rather a naming convention for the time between the identification and classification of a new disease.

The guidance applies to emerging infectious diseases that will occur in future and, not those that have already been identified and named.

For example, new disease names referring to places such as “Middle East Respiratory Syndrome” and “Spanish Flu”, or animals, such as “bird flu” and “monkey pox”, should be avoided. The use of people’s names, such as “Creutzfeldt-Jakob disease” and “Chagas disease” and occupations, such as “house-maid’s knee”, should also be avoided. 

Terms such as “unknown”, “death”, “fatal” and “epidemic” that may provoke undue fear should also not be part of new disease names, the guidance says.

“We’ve seen certain disease names provoke a backlash against members of particular religious or ethnic communities, create unjustified barriers to travel, commerce and trade, and trigger needless slaughtering of food animals,” said Dr Keiji Fukuda, WHO Assistant Director-General for Security.

“This can have serious consequences for peoples’ lives and livelihoods.”

The guidance, entitled *World Health Organization best practices for the naming of new human infectious diseases*, was developed in collaboration with the World Organisation for Animal Health and the Food and Agriculture Organization of the United Nations.

http://www.who.int/topics/infectious_diseases/naming-new-diseases/en/

**Looking ahead**

9–11 June – High-level International Conference on the Implementation of the Water for Life Decade. Dushanbe, Tajikistan. http://waterforlifeconf2015.org/eng/obosnovanie/

14 June – World Blood Donor Day. http://www.who.int/campaigns/world-blood-donor-day/2015/event/en/

13–16 July – 3rd International Conference on Financing for Development. Addis Ababa, Ethiopia. http://www.un.org/esa/ffd/overview/third-conference-ffd.html

28 July – World Hepatitis Day.

25 - 27 September – United Nations Summit to adopt the post-2015 development agenda. New York, USA.

